# Distortions of parliamentary amendments to the equitable allocation of federal resources to the PAB

**DOI:** 10.11606/s1518-8787.2022056004465

**Published:** 2022-11-18

**Authors:** Fabiola Sulpino Vieira, Luciana Dias de Lima

**Affiliations:** I Instituto de Pesquisa Econômica Aplicada Diretoria de Estudos e Políticas Sociais Brasília DF Brasil Instituto de Pesquisa Econômica Aplicada. Diretoria de Estudos e Políticas Sociais. Brasília, DF, Brasil; II Fundação Oswaldo Cruz Escola Nacional de Saúde Pública Sergio Arouca Departamento de Administração e Planejamento em Saúde Rio de Janeiro RJ Brasil Fundação Oswaldo Cruz. Escola Nacional de Saúde Pública Sergio Arouca. Departamento de Administração e Planejamento em Saúde. Rio de Janeiro, RJ, Brasil

**Keywords:** Health Care Rationing, legislation & jurisprudence, Unified Health System, Financing, Government, Public Expenditures on Health, Healthcare Disparities, economics

## Abstract

**OBJECTIVE:**

Analyze the implications of parliamentary amendments (EP) for the model of equitable allocation of resources from the Fixed Primary Care Minimum (PAB-Fixo) to municipalities in the period from 2015 to 2019.

**METHODS:**

A descriptive and exploratory study was conducted on allocating federal resources to the PAB-Fixo and on the increment in the PAB by parliamentary amendment. The municipalities were classified into four groups according to degrees of socioeconomic vulnerability defined by the Ministry of Health for the allocation of PAB-Fixo resources. The transfers from the Ministry by parliamentary amendment were identified. The proportions of municipalities benefiting per group were analyzed by resources allocated from the PAB-Fixo and increment to the minimum by EP.

**RESULTS:**

There were reduced resources allocated to the PAB-Fixo (from R$ 6.04 billion to R$ 5.51 billion, -8.8%) and increased increment to PAB by parliamentary amendment (from R$ 95.06 million to R$ 5.58 billion, 5.767%) between 2015 and 2019. The participation of municipalities by the group of those favored by EP was similar to that in the PAB-Fixo. In the proportion of resources for amendments, the municipalities of group I (most vulnerable) had more participation, and those of group IV had less participation if compared to the allocation of the PAB-Fixo. The distribution of resources by the parliamentary amendment did not cover all municipalities, even the most vulnerable ones, i.e., belonging to groups I and II. There was great inequality of resources *per capita* according to the groups of municipalities.

**CONCLUSION:**

The EP distorted the model of equitable allocation of resources proposed by the Ministry of Health for the PAB-Fixo, by allocating resources in a much more significant proportion to the municipalities of group I and much less to those of group IV, which is in disagreement with this model. Furthermore, this distribution by amendments does not benefit all municipalities, not even the most vulnerable.

## INTRODUCTION

In Brazil, the allocation of federal resources to states and municipalities through parliamentary amendments (EP) has been the object of analysis on public policies, with different approaches and focuses. Concerns about the political and decision-making process^1^and the effects of institutional rules on amendment distribution in the federal budget^4^ are highlighted.

In the case of resources allocated to the Brazilian Unified Health System (SUS), three facts contributed to placing the topic on the current agenda of discussion on health financing: increase in amounts allocated by individual amendments since the approval of the mandatory budget in 2015, which defines the obligation of its financial execution in the Federal Constitution of 1988; expansion of the execution of budget rapporteur amendments, which are non-compulsory, by the Ministry of Health (MS), and accounting of the amendments in the Ministry’s minimum expenditure on public health actions and services. Under the spending cap that freezes, in real terms, the minimum expenditure in public health actions and services of the Union, these facts favored the growth of the participation of the amendments, implying a reduction in the share of allocated resources, according to the Ministry of Health regulations^5^.

Studies indicate that EP can either contribute to the reduction of inequalities^6^ or ignore redistributive allocation criteria^7^, constituting more of an instrument for mediating relations between the Powers, aiming at the governability of the federal Executive^4^. This is an essential issue since direct transfers from the federal government to state and municipal governments in the Brazilian federative context are fundamental given the unequal availability of public services and resources from subnational entities^8^. In addition, specifically for the healthcare area, the Constitution establishes that the allocation of resources must have as a principle the progressive reduction of regional disparities in the country^9^.

Regarding the allocation of federal resources to primary healthcare (APS), before the significant increase in the execution of EP, the Ministry of Health had defined a method for equitably allocating amounts to the Fixed Primary Care Minimum (PAB-Fixo). The PAB-Fixo is, from the perspective of the municipalities, an essential source of resources for financing APS, in addition to being an instrument for allocating federal resources^4^. The values of the PAB-Fixo, added to other transfers, from the MS and state governments, in addition to own municipal resources, are used for the provision of actions and services at this level of healthcare in the SUS.

Transfers from the Ministry to municipalities, also called remittances, are carried out to finance specific interventions (transfer lines) and are organized in large areas of action of the healthcare system. In 2016, the median number of MS transfer lines for 5,569 municipalities was 22, i.e., half of the municipalities received 22 transfer lines. Of these, ten were for APS funding, one of which was PAB-Fixo^10^.

The method defined by the Ministry of Health for allocating resources to the PAB-Fixo considered socioeconomic indicators in constructing a vulnerability index that categorized municipalities into four groups for the *per capita* distribution of resources^11^. The model was in effect until 2019, when the MS created the Previne Brasil Program, establishing new funding criteria for primary healthcare in the SUS, starting in 2020^15^.

With increased parliamentary amendments’ participation in the APS financing, through a temporary increment in the PAB, there was an increased difference between the average values of the PAB-Fixo for the municipalities, according to their population size. Without the increment in the Primary Care Minimum, the difference between the averages of the *per capita* allocation of the municipalities was R$ 5.63 (24.8%) comparing the municipalities that received less and more resources in 2018. With the increment in the PAB, this difference was R$ 92.00 (367%)^5^.

These differences raise doubts about the consequences of allocating resources for a temporary increment in the Primary Care Minimum by EP and about the MS’s effort to allocate resources to municipalities within the scope of APS equitably. Were the municipalities favored by the EP the most vulnerable per the Ministry of Health’s categorization for the allocation of the PAB-Fixo? Did the EPs correspond in the distribution of resources with the allocation groups defined by the Ministry (proportion of beneficiaries per group and allocated amounts)?

Thus, this article aims to analyze the implications of EP for the model of equitable allocation of resources from the PAB-Fixo to municipalities established by the MS from 2015 to 2019. This approach is justified due to the topic’s relevance for discussion on health financing, the scarcity of scientific research that addresses EP to the SUS budget, and, more specifically, the lack of studies that answer the mentioned questions.

## METHODS

The descriptive and exploratory study conducted is based on the modern theory of the public budget, which defines it as a management instrument of i) political nature, as it expresses choices; ii ) economic, as it portrays the allocation of resources; iii ) managerial because it constitutes a plan, and iv ) legal because it is law^16^. Analyzes of public administration’s budget-financial execution make it possible to identify governments’ priorities in allocating resources, assess their planning and management capacity, and the compliance of their acts with budget laws^16,17^.

This article investigated the consequences of EP that increase resources to the Primary Care Minimum for the resource allocation model adopted by the MS for the PAB-Fixo^11^. In this model, the *per capita* transfers were defined according to a score from 0 to 10, calculated for each municipality, considering the following indicators: gross domestic product *per capita*, percentage of the population with health insurance, percentage of the population with *Bolsa Família*, percentage of the population in extreme poverty, and population density. The index created from these indicators reflects the degree of socioeconomic vulnerability of the population of each municipality, where zero indicates the maximum degree of vulnerability, i.e., worse socioeconomic conditions. The municipalities were classified into four groups, in a gradient of socioeconomic vulnerability, from highest to lowest:

- Group I: a score lower than 5.3 and a population of up to 50 thousand inhabitants – minimum of R$ 28.00 per inhabitant per year (inhab/year);
- Group II: scores between 5.3 and 5.8 and population of up to 100 thousand inhabitants or scores lower than 5.3 and population between 50 and 100 thousand inhabitants – minimum of R$ 26.00 inhab/year;
- Group III: scores between 5.8 and 6.1 and population of up to 500 thousand inhabitants or scores lower than 5.8 and population between 100 and 500 thousand inhabitants – minimum of R$ 24.00 inhab/year;
- Group IV: not included in the previous items and the Federal District (Brasília) – minimum of R$ 23.00 inhab/year.

Since the Ministry of Health did not publish the list of municipalities by group, the classification of each of them had to be inferred from the division between the annual PAB-Fixo value by the 2012 reference population for the period from 2015 to 2017 (Annex II of Ordinance MS/GM No. 1,409, of 2013)^12^, and between the value of the annual PAB-Fixo by the reference population of 2016, for 2018 and 2019 (Annex II of Ordinance MS/GM No. 3,947, of 2017)^13^, thus obtaining the annual *per capita transfer* value. The assumption was made that the values published in the ordinances result from applying the criteria and methods adopted by the Ministry for the equitable allocation of resources in the PAB-Fixo.

Two MS determinations regarding the transfers of the PAB-Fixo were analyzed for the possible impact on the groups’ inference. The first is that the Ministry established that the municipalities would not suffer a reduction in the value of the PAB-Fixo due to population variation. Comparing the transferred amount contained in the transfer file of the National Health Fund (FNS) of 2018 concerning the MS/GM Ordinance No. 3,947^13^ showed that the FNS transfer of the 455 municipalities that had a reduction in the reference population (2016 compared to 2012), compared to the ordinance, was higher for 450 municipalities and the same for five of them. Therefore, the values of the ordinance do not seem to contain adjustments due to population reduction.

The second is that, in 2013, the MS decided to integrate the values of the Compensation of Regional Specificities (CER) strategy of the PAB-Variável to the PAB-Fixo^11,18^. As a result, it became more complicated to reproduce the comparison mentioned above for cases of reduction in the reference population (2012 compared to 2010)^13^. However, when comparing the *per capita* value calculated from the annual value of the PAB-Fixo of Ordinance MS/GM No. 1,409 and that obtained from the FNS transfer files, including the CER strategy, greater consistency is observed in the first case, with values greater than R$ 23.00 *per capita*/year. The same does not occur when information from the transfer file of the National Health Fund is used, as transfer values lower than this minimum are obtained. This result indicates that the value of the ordinance encompasses the entire value of the CER strategy, and its use in the inference of groups is more appropriate.

The increment values in the PAB transferred by the Ministry of Health to each municipality were obtained from the FNS transfer files. This increment concerns federal resources allocated by EP^5^. For comparison in the analyzed period, the resources destined for the PAB-Fixo and the increment in the Primary Care Minimum were monetarily updated for 2020, using the average annual Broad Consumer Price Index (IPCA).

The data were organized in electronic spreadsheets and summarized with basic descriptive statistics. The Z test was applied with the support of the RStudio software, version 2021.09.0, considering a 95% confidence interval (95%CI) to compare the proportions of municipalities and resources between the PAB and its increment in the four groups^19^.

## RESULTS

In 2015, 83.6% of the municipalities (n = 5,570) were classified in groups I (n = 2,604) and II (n = 2,051) in terms of PAB-Fixo (Table 1 and Table 2). In 2019, this percentage grew (to 91.3%), with 3,958 municipalities in group I and 1,130 in group II.

**Table 1:** Municipalities favored by parliamentary amendments for the increment to the Primary Care Minimum (PAB) between 2015 and 2019, according to large regions and PAB-Fixo resource allocation groups defined by the Ministry of Health.

Regions	PAB-Fixo resource allocation groups								Total	

	I		II		III		IV			
				
	n	Group (%)	n	Group (%)	n	Group (%)	n	Group (%)	n	Group (%)
2015, 2016, and 2017 - All municipalities (PAB-Fixo)										
CW	273	10.5	162	7.9	27	3.6	5	3.0	467	8.4
NE	1,079	41.4	621	30.3	81	10.8	13	7.9	1,794	32.2
N	261	10.0	152	7.4	34	4.5	3	1.8	450	8.1
SE	617	23.7	600	29.3	348	46.3	103	62.8	1,668	29.9
S	374	14.4	516	25.2	261	34.8	40	24.4	1,191	21.4
**Total**	**2,604**	**100.0**	**2,051**	**100.0**	**751**	**100.0**	**164**	**100.0**	**5,570**	**100.0**
2015 - Municipalities benefited from the increment in the PAB by parliamentary amendments										
CW	0	0.0	0	0.0	0	0.0	0	0.0	0	0.0
NE	49	35.3	30	26.3	4	7.7	2	22.2	85	27.1
N	16	11.5	15	13.2	3	5.8	0	0.0	34	10.8
SE	42	30.2	30	26.3	26	50.0	5	55.6	103	32.8
S	32	23.0	39	34.2	19	36.5	2	22.2	92	29.3
**Total**	**139**	**100.0**	**114**	**100.0**	**52**	**100.0**	**9**	**100.0**	**314**	**100.0**
2016 - Municipalities benefited from the increment in the PAB by parliamentary amendments										
CW	14	1.6	4	0.6	4	1.2	0	0.0	22	1.1
NE	361	41.2	188	25.9	36	11.2	4	8.2	589	29.9
N	78	8.9	49	6.8	12	3.7	1	2.0	140	7.1
SE	231	26.3	215	29.7	135	42.1	30	61.2	611	31.0
S	193	22.0	269	37.1	134	41.7	14	28.6	610	30.9
**Total**	**877**	**100.0**	**725**	**100.0**	**321**	**100.0**	**49**	**100.0**	**1,972**	**100.0**
2017 - Municipalities benefited from the increment in the PAB by parliamentary amendments										
CW	104	6.2	60	4.4	8	1.6	2	2.2	174	4.8
NE	699	42.0	428	31.4	54	10.8	8	8.8	1,189	32.8
N	203	12.2	112	8.2	16	3.2	1	1.1	332	9.2
SE	394	23.7	391	28.7	229	45.7	54	59.3	1,068	29.5
S	265	15.9	372	27.3	194	38.7	26	28.6	857	23.7
**Total**	**1,665**	**100.0**	**1,363**	**100.0**	**501**	**100.0**	**91**	**100.0**	**3,620**	**100.0**
2018 and 2019 - All municipalities (PAB-Fixo)										
CW	411	10.4	33	2.9	18	4.6	5	5.3	467	8.4
NE	1,608	40.6	123	10.9	52	13.4	11	11.7	1,794	32.2
N	379	9.6	44	3.9	23	5.9	4	4.3	450	8.1
SE	957	24.2	464	41.1	190	49.0	57	60.6	1,668	29.9
S	603	15.2	466	41.2	105	27.1	17	18.1	1,191	21.4
**Total**	**3,958**	**100.0**	**1,130**	**100.0**	**388**	**100.0**	**94**	**100.0**	**5,570**	**100.0**
2018 - Municipalities benefited from the increment in the PAB by parliamentary amendments										
CW	298	8.6	21	2.1	9	3.0	1	1.5	329	6.8
NE	1,455	42.0	111	11.2	44	14.5	7	10.4	1,617	33.5
N	359	10.4	36	3.6	16	5.3	3	4.5	414	8.6
SE	809	23.3	381	38.6	146	48.2	40	59.7	1,376	28.5
S	544	15.7	439	44.4	88	29.0	16	23.9	1,087	22.5
**Total**	**3,465**	**100.0**	**988**	**100.0**	**303**	**100.0**	**67**	**100.0**	**4,823**	**100.0**
2019 - Municipalities benefited from the increment in the PAB by parliamentary amendments										
CW	367	10.0	29	2.8	14	3.9	2	2.3	412	8.0
NE	1,554	42.6	117	11.3	49	13.7	11	12.5	1,731	33.7
N	369	10.1	42	4.1	20	5.6	4	4.5	435	8.5
SE	824	22.6	410	39.7	173	48.3	56	63.6	1,463	28.5
S	538	14.7	434	42.1	102	28.5	15	17.0	1,089	21.2
**Total**	**3,652**	**100.0**	**1,032**	**100.0**	**358**	**100.0**	**88**	**100.0**	**5,130**	**100.0**

Sources: Brasil (2013; 2017)^12,13^ and the National Health Fund (FNS). Transfer files. Available from: <https://portalfns.saude.gov.br/downloads/>.

**Table 2:** Municipalities favored by parliamentary amendments for the increment to the Primary Care Minimum (PAB) between 2015 and 2019, according to population size ranges and PAB-Fixo resource allocation groups defined by the Ministry of Health.

Population size ranges	PAB-Fixo resource allocation groups								Total	

	I		II		III		IV			
				
	n	Group (%)	n	Group (%)	n	Group (%)	n	Group (%)	n	Group (%)
2015, 2016, and 2017 – All municipalities (PAB-Fixo)										
≤ 5,000	694	26.7	508	24.8	90	12.0	8	4.9	1,300	23.3
5,001–10,000	670	25.7	434	21.2	96	12.8	11	6.7	1,211	21.7
10,001–20,000	755	29.0	508	24.8	121	16.1	8	4.9	1,392	25.0
20,001–50,000	485	18.6	416	20.3	126	16.8	28	17.1	1,055	18.9
50,001–100,000	0	0.0	185	9.0	126	16.8	13	7.9	324	5.8
100,001–500,000	0	0.0	0	0.0	192	25.6	58	35.4	250	4.5
≥ 500,000	0	0.0	0	0.0	0	0.0	38	23.2	38	0.7
**Total**	**2,604**	**100.0**	**2,051**	**100.0**	**751**	**100.0**	**164**	**100.0**	**5,570**	**100.0**
2015 – Municipalities benefited from the increment in the PAB by parliamentary amendments										
≤ 5,000	36	25.9	22	19.3	4	7.7	0	0.0	62	19.7
5,001–10,000	43	30.9	27	23.7	10	19.2	0	0.0	80	25.5
10,001–20,000	33	23.7	32	28.1	10	19.2	0	0.0	75	23.9
20,001–50,000	27	19.4	26	22.8	10	19.2	3	33.3	66	21.0
50,001–100,000	0	0.0	7	6.1	7	13.5	0	0.0	14	4.5
100,001–500,000	0	0.0	0	0.0	11	21.2	2	22.2	13	4.1
≥ 500,000	0	0.0	0	0.0	0	0.0	4	44.4	4	1.3
**Total**	**139**	**100.0**	**114**	**100.0**	**52**	**100.0**	**9**	**100.0**	**314**	**100.0**
2016 – Municipalities benefited from the increment in the PAB by parliamentary amendments										
≤ 5,000	221	25.2	151	20.8	32	10.0	0	0.0	404	20.5
5,001–10,000	232	26.5	157	21.7	44	13.7	3	6.1	436	22.1
10,001–20,000	250	28.5	195	26.9	59	18.4	1	2.0	505	25.6
20,001–50,000	174	19.8	161	22.2	55	17.1	10	20.4	400	20.3
50,001–100,000	0	0.0	61	8.4	58	18.1	5	10.2	124	6.3
100,001–500,000	0	0.0	0	0.0	73	22.7	16	32.7	89	4.5
≥ 500,000	0	0.0	0	0.0	0	0.0	14	28.6	14	0.7
**Total**	**877**	**100.0**	**725**	**100.0**	**321**	**100.0**	**49**	**100.0**	**1972**	**100.0**
2017 – Municipalities benefited from the increment in the PAB by parliamentary amendments										
≤ 5,000	485	29.1	337	24.7	62	12.4	5	5.5	889	24.6
5,001–10,000	454	27.3	289	21.2	72	14.4	7	7.7	822	22.7
10,001–20,000	457	27.4	337	24.7	94	18.8	3	3.3	891	24.6
20,001–50,000	269	16.2	274	20.1	84	16.8	18	19.8	645	17.8
50,001–100,000	0	0.0	126	9.2	84	16.8	7	7.7	217	6.0
100,001–500,000	0	0.0	0	0.0	105	21.0	29	31.9	134	3.7
≥ 500,000	0	0.0	0	0.0	0	0.0	22	24.2	22	0.6
**Total**	**1,665**	**100.0**	**1,363**	**100.0**	**501**	**100.0**	**91**	**100.0**	**3,620**	**100.0**
2018 and 2019 – All municipalities (receiving the PAB-Fixo)										
≤ 5,000	1,052	26.6	166	14.7	16	4.1	3	3.2	1,237	22.2
5,001–10,000	1,018	25.7	169	15.0	22	5.7	0	0.0	1,209	21.7
10,001–20,000	1,096	27.7	246	21.8	17	4.4	5	5.3	1,364	24.5
20,001–50,000	792	20.0	248	21.9	52	13.4	9	9.6	1,101	19.8
50,001–100,000	0	0.0	301	26.6	43	11.1	6	6.4	350	6.3
100,001–500,000	0	0.0	0	0.0	238	61.3	30	31.9	268	4.8
≥ 500,000	0	0.0	0	0.0	0	0.0	41	43.6	41	0.7
**Total**	**3,958**	**100.0**	**1,130**	**100.0**	**388**	**100.0**	**94**	**100.0**	**5,570**	**100.0**
2018 – Municipalities benefited from the increment in the PAB by parliamentary amendments										
≤ 5,000	908	26.2	147	14.9	12	4.0	3	4.5	1,070	22.2
5,001–10,000	907	26.2	155	15.7	18	5.9	0	0.0	1,080	22.4
10,001–20,000	963	27.8	229	23.2	16	5.3	3	4.5	1,211	25.1
20,001–50,000	687	19.8	202	20.4	41	13.5	9	13.4	939	19.5
50,001–100,000	0	0.0	255	25.8	35	11.6	4	6.0	294	6.1
100,001–500,000	0	0.0	0	0.0	181	59.7	23	34.3	204	4.2
≥ 500,000	0	0.0	0	0.0	0	0.0	25	37.3	25	0.5
**Total**	**3,465**	**100.0**	**988**	**100.0**	**303**	**100.0**	**67**	**100.0**	**4,823**	**100.0**
2019 – Municipalities benefited from the increment in the PAB by parliamentary amendments										
≤ 5,000	931	25.5	143	13.9	13	3.6	2	2.3	1,089	21.2
5,001–10,000	935	25.6	149	14.4	17	4.7	0	0.0	1,101	21.5
10,001–20,000	1,042	28.5	225	21.8	16	4.5	4	4.5	1,287	25.1
20,001–50,000	744	20.4	230	22.3	50	14.0	9	10.2	1,033	20.1
50,001–100,000	0	0.0	285	27.6	41	11.5	6	6.8	332	6.5
100,001–500,000	0	0.0	0	0.0	221	61.7	29	33.0	250	4.9
≥ 500,000	0	0.0	0	0.0	0	0.0	38	43.2	38	0.7
**Total**	**3,652**	**100.0**	**1,032**	**100.0**	**358**	**100.0**	**88**	**100.0**	**5,130**	**100.0**

Sources: Brasil (2013; 2017)^12,13^ and the National Health Fund (FNS). Transfer files. Available from: <https://portalfns.saude.gov.br/downloads/>.

In group 1, with greater socioeconomic vulnerability, more than half of the municipalities belong to the Central-West and Northeast regions, with a population equal to or less than 50 thousand inhabitants (PAB-Fixo 2015 reference) (Table 1). Regarding the municipalities benefiting from EP, between 2015 and 2017, the proportion of those favored in these regions was below 50% for members of group I, with municipalities in the Southeast and South regions being more prevalent. In 2018 and 2019, there was a greater balance between the proportion of beneficiaries per group and region. This occurred for the PAB-Fixo and the increment in the PAB.

By population size, the allocation of resources by EP was close to that defined for the Primary Care Minimum in the case of group I (Table 2). In 2015, 10.5% of municipalities with over 500 thousand inhabitants and 4.8% with up to 5 thousand inhabitants were favored by EP. In 2017 and 2019, these percentages increased to 57.6% and 68.4%, and to 92.7% and 88.0%, respectively.

Between 2015 (value valid until 2017) and 2019 (value valid in 2018 and 2019), there was a reduction in the resources allocated to the PAB-Fixo, in real terms (Table 3). The PAB-Fixo went from R$ 6.04 billion to R$ 5.51 billion at 2020 prices (-8.8%). In the same period, the increment in the PAB went from R$ 95.06 million to R$ 5.58 billion, with a growth of 5,767%.

**Table 3:** Federal transfers from PAB-Fixo to the municipalities and for incrementing the PAB through parliamentary amendments, according to the PAB-Fixo resource allocation groups defined by the Ministry of Health.

Resource Allocation Groups	PAB-Fixo	Increment to the PAB			PAB-Fixo	Increment to the PAB	
			
	2015, 2016, and 2017	2015	2016	2017	2018 and 2019	2018	2019
Total amount in 2020 R$							
Group I	1,124,748,593	34,042,736	348,535,796	888,855,288	1,501,737,158	2,806,017,353	3,159,522,314
Group II	1,242,968,275	32,436,990	292,125,244	760,961,497	943,886,451	833,223,227	1,104,026,427
Group III	1,594,723,442	19,670,932	212,915,263	381,464,102	1,317,320,727	509,097,654	888,507,783
Group IV	2,078,489,331	8,908,991	84,636,888	113,825,593	1,745,813,233	190,696,645	424,829,357
**Brazil**	**6,040,929,641**	**95,059,649**	**938,213,191**	**2,145,106,481**	**5,508,757,569**	**4,339,034,880**	**5,576,885,881**
Average *per capita* value in 2020 R$							
Group I	35	31	44	61	30	81	80
Group II	34	27	36	50	28	45	46
Group III	31	13	21	30	26	18	23
Group IV	29	6	8	15	25	9	15
**Brazil**	**34**	**26**	**36**	**52**	**29**	**69**	**68**
Percentage of municipalities benefited by group (%)							
Group I	100	5	34	64	100	88	92
Group II	100	6	35	66	100	87	91
Group III	100	7	43	67	100	78	92
Group IV	100	5	30	55	100	71	94
**Brazil**	**100**	**6**	**35**	**65**	**100**	**87**	**92**

PAB: Primary Care Minimum.

Sources: Brasil (2013; 2017)^12,13^ and the National Health Fund (FNS). Transfer files. Available from: <https://portalfns.saude.gov.br/downloads/>.

Values monetarily updated by the average annual IPCA.

In 2017, the increment was equivalent to 35.5% of PAB-Fixo resources (R$ 2.15 billion in R$ 6.04 billion). In 2019, this percentage was 101.2% (R$ 5.58 billion in R$ 5.51 billion). Of the amount allocated by EP (increment) in 2015, 69.9% was allocated to groups I and II (R$ 66.5 million in R$ 95.06 million). In 2019, they increased to 76.5% for the same groups (R$ 4.26 billion in R$ 5.58 billion).

Per Table 3, in 2015, 5% of the municipalities in group I benefited, on average, with R$ 31.00 *per capita* (at 2020 prices) incrementing to PAB. They received R$ 35.00 *per capita* from the PAB-Fixo plus R$ 31.00 *per capita* for EP. In the same year, 95% of the municipalities in this group had only R$ 35.00 *per capita* from the PAB-Fixo. In 2019, 92% of the municipalities in group I were favored by EP and had an additional Primary Care Minimum of R$ 80.00 *per capita* on average, while 8% of the municipalities in this group had only the PAB-Fixo (R$ 30.00 *per capita*).

Table 4 compares the proportions of municipalities and resources allocated to the PAB-Fixo classes and increment to the PAB by groups. It can be seen that the null hypothesis of equality between the classes regarding the proportions of municipalities cannot be rejected since the *p-value* is greater than 0.05. Thus, for the group of EP beneficiaries, the participation of municipalities per group was similar to the participation of municipalities in the PAB-Fixo per group.

**Table 4:** Proportion of municipalities and resources concerning the PAB-Fixo and incremented PAB from 2015 to 2019, according to the PAB-Fixo resource allocation groups.

Resource allocation groups	Base of comparison 2015				Base of comparison 2018			
	
	PAB-Fixo	Increment to PAB	Z-test		PAB-Fixo	Increment to PAB	Z-test	
					
	2015, 2016, and 2017	Average 2015–2017	X2	p	2018 and 2019	Average 2018–2019	X2	p
Proportion of municipalities								
Group I	47	45	0.02	0.88720	71	72	8.86	1.00000
Group II	37	37	0.00	1.00000	20	20	0.00	1.00000
Group III	13	16	0.16	0.68790	7	7	0.00	1.00000
Group IV	3	3	0.00	1.00000	2	2	0.00	1.00000
**Total**	**100**	**100**			**100**	**100**		
Proportion of resources								
Group I	19	38	7.95	0.00481^b^	27	61	22.10	0.002591^b^
Group II	21	34	3.61	0.05739	17	19	0.03	0.85400
Group III	26	20	0.71	0.40080	24	14	2.63	0.10480
Group IV	34	8	18.84	0.00001^a^	32	6	20.30	0.006601^b^
**Total**	**100**	**100**			**100**	**100**		

PAB: Primary Care Minimum.

Sources: Brasil (2013; 2017)^12,13^ and the National Health Fund (FNS). Transfer files. Available from: <https://portalfns.saude.gov.br/downloads/>.

^a^ p-value < 0.001 (statistical significance at 0.1%);

^b^ p-value < 0.01 (statistical significance at 1% level).

However, concerning the proportions of resources, a statistically significant *p-value* is observed at the 0.1% level, indicating that the 2015 PAB-Fixo classes and the average of the 2015–2017 increment are different for group IV, and at the 1% level for group I of these same classes. Likewise, for groups I and IV of the 2018 PAB-Fixo classes and the average of the 2018–2019 increment. In other words, in the resources allocated by EP, the municipalities of group I had greater participation, and those of group IV had lower participation compared to the participation of these groups in PAB-Fixo resources.

There is a significant increase in the PAB-Fixo plus the increment in the PAB from 2017 (Figure). When comparing the groups, for the municipalities in group I, the average per capita value of the PAB-Fixo with increments went from R$ 66.00 to R$ 108.00 (64.3%) between 2015 and 2019. For group IV, the increase was 11.8%, from R$ 35.00 to R$ 39.00 in the same period.

**Figure:**
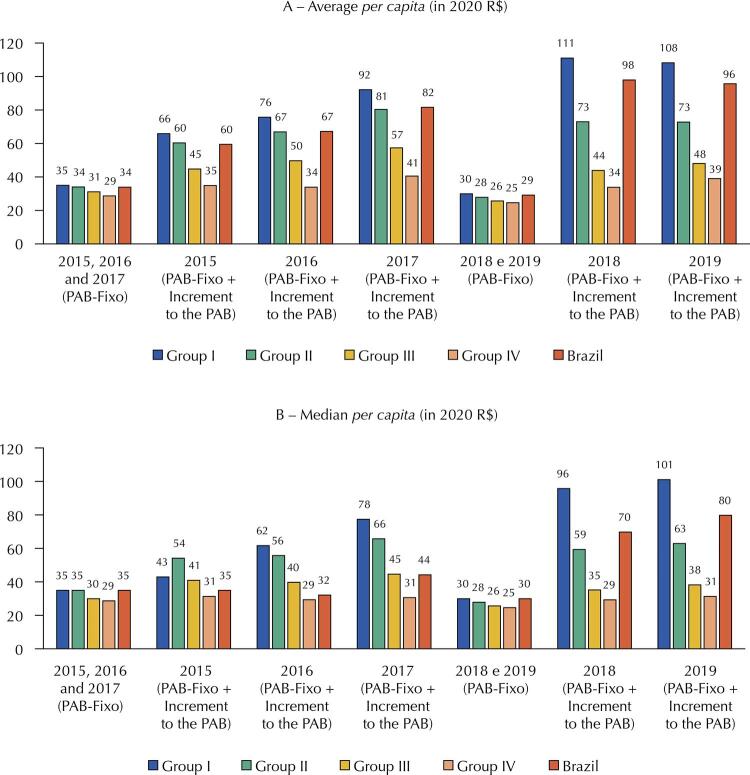
Average and median of the Primary Care Minimum-Fixed (PAB-Fixo) *per capita* and the PAB-Fixo plus the increment to the *per capita* PAB, according to the PAB-Fixo resource allocation groups to the municipalities, defined by the Ministry of Health. Brasil, 2015–2019.

Sources: Brasil (2013; 2017)^12,13^ and the National Health Fund (FNS). Transfer files. Available from: <https://portalfns.saude.gov.br/downloads/>.

Own elaboration.

The Figure also shows that the averages and medians of the PAB-Fixo present low variation, which does not occur when the increment values are added. This indicates that there are municipalities with a very high increment in the PAB *per capita*, which causes a greater distance between the average and median of the analyzed values. Finally, the reduction, in real terms, of the averages and medians *per capita* of the PAB-Fixo stands out. On average, considering all municipalities, it went from R$ 34.00 in 2015 to R$ 29.00 in 2019 (-14.7%).

## DISCUSSION

Some methodologies have been developed within the scope of health systems for the equitable allocation of resources^20^. In this sense, different meanings of equity have been used, such as i) the complete equalization of opportunities to access the same number of services concerning needs, ii) ensuring that no particular group is disadvantaged, and iii) everyone has an equal opportunity to lead a healthy life^21^.

Equity is also cited as a fair opportunity for all, equal access to health services based on needs, and the absence of systematic health inequalities between socioeconomically different groups, reported in the literature as the most used criterion by decision-makers on healthcare allocation of resources^22^.

In Brazil, the idea of equitable allocation of resources is intrinsically associated with health needs. In general, while the authors defend centrality and recognize the complex form of the concept of health needs for allocating resources, they do not make it explicit. However, it is possible to assume its reach beyond the system’s borders due to using socioeconomic, demographic, and health indicators in the methodologies proposed or analyzed^23^.

Methodologies for the equitable allocation of resources generally reflect the idea that it is necessary to consider the unequal living conditions of the population to allocate resources unequally. The purpose is to allocate more resources to the most disadvantaged groups from demographic, social, economic, and health points of view.

In the SUS, implementing the PAB-Fixo is among the initiatives adopted by the MS to promote the reduction of regional inequalities through an unequal allocation of federal resources for health^26^. Although the initiative may eventually be criticized regarding the method adopted and the results obtained, its merits cannot be ignored when introducing the idea of equitable allocation of resources in the SUS. This system presents a pattern of shared responsibility among the entities in financing primary care services. However, it is up to the Ministry of Health to distribute financial resources to compensate for inequalities between municipalities, mainly responsible for managing these services^9^.

This study demonstrates that, in terms of favored municipalities, the EP followed the allocation groups defined by the MS, benefiting them in a similar proportion to the distribution made for the PAB-Fixo. However, the PAB-Fixo favors all municipalities, unlike the EP, even though its coverage has increased in the period analyzed.

Concerning the PAB-Fixo allocation model, the analysis of the allocated resources shows that the resources of EP were allocated in more significant proportions to the municipalities of group I and lesser proportions to those of group IV. In other words, municipalities with a population of up to 50 thousand inhabitants, more socioeconomically vulnerable, were prioritized with the contributions by EP, to the detriment of less vulnerable municipalities, with a population above 50 thousand inhabitants.

As a result, there is a greater distance between the *per capita* values of the PAB when the increment resources are added. Populations of smaller municipalities are benefiting from much more resources for APS financing than those of larger, less vulnerable municipalities.

In principle, such a situation is desirable concerning equitable allocation. However, it is necessary to consider the current situation of SUS financing and the possible impacts of the allocation of resources by EP, given the considerable constraint imposed on the Ministry budget by the spending cap for primary federal expenditures and the freezing of the federal minimum application in public health actions and services^27^. As expenses for EP are accounted for in the minimum application, greater allocation of resources by EP reduces the share of the Ministry of Health’s allocation in actions and services. It may imply the reallocation of resources from other areas. In the case analyzed, from EP to increment to the PAB.

It is also important to highlight that even among the municipalities in group I, those benefiting from EP received much more *per capita* resources than those not. As a result, the amendments generated unequal treatment among the most vulnerable.

The very different *per capita* value between the groups of municipalities implies a differentiated benefit among their populations. Public health actions and services must be guaranteed in all municipalities, and in smaller ones, the costs of offering them are usually higher^28^. However, it is necessary to remember that there are difficulties in structuring them on the outskirts of medium and large-sized cities^29^. The financial crisis from 2014 to 2016, and more recently, the impacts of the pandemic on the Brazilian economy, caused a drop in municipal revenue and, thus, more significant difficulties in allocating resources to health. Municipalities already apply them far above the mandatory minimum percentage^30^.

Ultimately, an allocation of federal resources that does not consider the differentiated fiscal capacity of entities can also cause inequity, even if its objective is equity. The adoption of technical criteria has been identified as necessary to mitigate this problem^31^. This issue needs to be deepened in future studies for the case of EP in general.

Other issues that must be considered concerning the use of EP to guarantee the support base of the federal government, in the National Congress, in an unprecedented way, considering the high number of resources involved and the massive lack of transparency in its execution. This lack, especially of the rapporteur’s parliamentary amendments, was questioned in the Federal Supreme Court (STF), which ordered Congress to publish the list of favored members and parliamentarians in addition to the amounts allocated^32^.

The consequences of an allocation of resources that considers strictly political criteria can be very harmful to the SUS, given the context of budget constraint already mentioned. In addition to causing more significant inequalities, it can be more inefficient, which is unacceptable given the limited resources for financing public health in the country.

This work points out the inference of resource allocation groups as a limitation, which generates some uncertainty about the category of each municipality. The lack of transparency about the Ministry of Health’s method constitutes a barrier not only to the knowledge of the classification of these entities but also to any study aiming to investigate this initiative of equitable allocation of resources.

Finally, it should be noted that only the implications of the EP on the model adopted by the Ministry for the PAB-Fixo were analyzed. The model itself has not been evaluated. In conclusion, the parliamentary amendments distorted the model of equitable allocation of resources thought by the MS for the PAB-Fixo, by allocating resources in a much more significant proportion to the municipalities of group I and much less to those of group IV, in disagreement with this model, and for not benefiting all municipalities, not even the most vulnerable.
