# Supplementary material for: Distortions of parliamentary amendments to the equitable allocation of federal resources to the PAB
Source: Rev Saude Publica. 2022 Nov 18;56:123. doi: 10.11606/s1518-8787.2022056004465 (PMC9749733; doi:10.11606/s1518-8787.2022056004465)
Supplement: Translation

# Translation: Distorções das emendas parlamentares à alocação equitativa de recursos federais ao PAB

**Authors:** Fabiola Sulpino Vieira, Luciana Dias de Lima

**Affiliations:** I Brasil Instituto de Pesquisa Econômica Aplicada. Diretoria de Estudos e Políticas Sociais. Brasília, DF, Brasil; II Brasil Fundação Oswaldo Cruz. Escola Nacional de Saúde Pública Sergio Arouca. Departamento de Administração e Planejamento em Saúde. Rio de Janeiro, RJ, Brasil

**Keywords:** Alocação de Recursos para a Atenção à Saúde, legislação & jurisprudência, Sistema Único de Saúde, Financiamento Governamental, Gastos Públicos com Saúde, Disparidades em Assistência à Saúde, economia

## Abstract

**OBJETIVO:**

Analisar as implicações das emendas parlamentares (EP) para o modelo de alocação equitativa de recursos do Piso da Atenção Básica Fixo (PAB-Fixo) aos municípios no período de 2015 a 2019.

**MÉTODOS:**

Realizou-se um estudo descritivo e exploratório da alocação de recursos federais para o PAB-Fixo e para incremento ao PAB por emenda parlamentar. Os municípios foram classificados em quatro grupos, segundo graus de vulnerabilidade socioeconômica definidos pelo Ministério da Saúde para destinação de recursos do PAB-Fixo. Os repasses do ministério por emenda parlamentar foram identificados, analisando-se as proporções de municípios beneficiados em cada grupo por recursos alocados do PAB-Fixo e do incremento ao piso por EP.

**RESULTADOS:**

Verificou-se redução dos recursos alocados ao PAB-Fixo (de R$ 6,04 bilhões para R$ 5,51 bilhões, -8,8%) e aumento do incremento ao PAB por emenda parlamentar (de R$ 95,06 milhões para R$ 5,58 bilhões, 5.767%) entre 2015 e 2019. A participação dos municípios por grupo dos que foram favorecidos por EP foi semelhante à dos municípios do PAB-Fixo. Na proporção de recursos por emendas, os municípios do grupo I (mais vulneráveis) tiveram maior participação e os do grupo IV, menor participação, se comparada à alocação do PAB-Fixo. A distribuição de recursos por emenda parlamentar não contemplou todos os municípios, mesmo aqueles mais vulneráveis, pertencentes aos grupos I e II. Houve grande desigualdade de recursos *per capita* segundo os grupos de municípios.

**CONCLUSÃO:**

As EP distorceram o modelo de alocação equitativa de recursos proposto pelo Ministério da Saúde para o PAB-Fixo, ao destinar recursos em proporção muito maior para os municípios do grupo I e muito menor para os do grupo IV, o que está em desacordo com esse modelo, além disso essa distribuição por emendas não beneficia a todos os municípios, nem mesmo aos mais vulneráveis.

## INTRODUÇÃO

No Brasil, a destinação de recursos federais a estados e municípios por meio de emendas parlamentares (EP) tem sido objeto de análises sobre políticas públicas, com abordagens e enfoques diversos. Destacam-se preocupações quanto ao processo político e decisório^1^ e aos efeitos das regras institucionais na distribuição das emendas no orçamento federal^4^.

No caso dos recursos alocados ao Sistema Único de Saúde (SUS), três fatos contribuíram para colocar o tema na agenda atual de discussão sobre o financiamento da saúde: aumento dos montantes alocados por emendas individuais, desde a aprovação do orçamento impositivo, em 2015, que define a obrigatoriedade de sua execução financeira na Constituição Federal de 1988; ampliação da execução de emendas de relator do orçamento, que são de execução não compulsória, pelo Ministério da Saúde (MS); e contabilização das emendas no gasto mínimo do ministério em ações e serviços públicos de saúde. Na vigência do teto de gastos que congela, em termos reais, a aplicação mínima em ações e serviços públicos de saúde da União, esses fatos favoreceram o crescimento da participação das emendas, implicando redução da parcela de recursos alocados, segundo normatização do Ministério da Saúde^5^.

Estudos indicam que as EP tanto podem contribuir para a redução das desigualdades^6^, como podem ignorar critérios redistributivos de alocação^7^, constituindo-se mais em instrumento de mediação das relações entre os Poderes, visando à governabilidade do Executivo federal^4^. Essa é uma questão importante, uma vez que, no contexto federativo brasileiro, as transferências diretas do governo federal para os governos estaduais e municipais são fundamentais diante da desigual disponibilidade de oferta de serviços públicos e de recursos dos entes subnacionais^8^. Além disso, especificamente para a área da saúde, a Constituição estabelece que a alocação de recursos deve ter por princípio a progressiva diminuição das disparidades regionais no país^9^.

Em relação à destinação de recursos federais para a atenção primária à saúde (APS), antes do aumento significativo da execução das EP, o Ministério da Saúde havia definido um método para alocação equitativa dos valores ao Piso da Atenção Básica Fixo (PAB-Fixo). O PAB-Fixo, além de instrumento para alocação de recursos federais, constitui, sob a perspectiva dos municípios, importante fonte de recursos para o financiamento da APS^4^. Os valores do PAB-Fixo, somados aos de outras transferências, do MS e dos governos estaduais, além dos recursos próprios municipais, são utilizados para a provisão de ações e serviços nesse nível de atenção à saúde no SUS.

As transferências do ministério aos municípios, também chamadas de repasses, são realizadas para financiamento de intervenções específicas (linhas de repasse), e organizadas em grandes áreas de atuação do sistema de saúde. Em 2016, a mediana do número de linhas de repasse do MS para 5.569 municípios foi de 22, ou seja, metade dos municípios recebeu 22 linhas de repasse. Destas, 10 foram para financiamento da APS, sendo uma delas o PAB-Fixo^10^.

O método definido pelo Ministério da Saúde para alocação de recursos ao PAB-Fixo considerava indicadores socioeconômicos na construção de um índice de vulnerabilidade que categorizava os municípios em quatro grupos para a distribuição *per capita* dos recursos^11^. O modelo vigorou até o exercício de 2019, quando o MS criou o Programa Previne Brasil, estabelecendo novos critérios de financiamento de custeio para a atenção primária à saúde no SUS, a partir de 2020^15^.

Com o aumento da participação das EP no custeio da APS, por meio de dispositivo de incremento temporário ao PAB, verificou-se ampliação da diferença entre os valores médios do PAB-Fixo para os municípios, segundo seu porte populacional. Sem o incremento ao Piso da Atenção Básica, a diferença entre as médias da alocação *per capita* dos municípios foi de R$ 5,63 (24,8%) entre os municípios que recebiam menos e mais recursos em 2018. Com o incremento ao PAB, essa diferença foi de R$ 92,00 (367%)^5^.

Essas diferenças suscitam dúvidas sobre as consequências da alocação de recursos para incremento temporário ao Piso da Atenção Básica por EP e sobre o esforço do MS de alocação equitativa de recursos aos municípios no âmbito da APS. Os municípios favorecidos pelas EP eram os mais vulneráveis de acordo com a categorização feita pelo Ministério da Saúde para alocação do PAB-Fixo? As EP guardaram correspondência na distribuição de recursos com os grupos de alocação definidos pelo ministério (proporção de favorecidos por grupo e dos valores alocados)?

Assim, o objetivo deste artigo é analisar as implicações das EP para o modelo de alocação equitativa de recursos do PAB-Fixo aos municípios instituído pelo MS, no período de 2015 a 2019. A justificativa para esse recorte se deve à relevância do tema para a discussão sobre o financiamento da saúde, à escassez de pesquisas científicas que tratem das EP ao orçamento do SUS e, mais especificamente, à falta de estudos que respondam as questões mencionadas.

## MÉTODOS

O estudo descritivo e exploratório realizado se baseia na teoria moderna do orçamento público, que o define como instrumento de gestão de natureza: i) política, por expressar escolhas; ii) econômica, por retratar a alocação de recursos; iii) gerencial, porque constitui um plano; e iv) jurídica, porque é lei^16^. As análises sobre a execução orçamentário-financeira da administração pública possibilitam identificar as prioridades dos governos na alocação dos recursos, avaliar a sua capacidade de planejamento e gestão, e a conformidade de seus atos às leis orçamentárias^16,17^.

Neste artigo, investigaram-se as consequências das EP que incrementam recursos ao Piso da Atenção Básica para o modelo de alocação de recursos adotado pelo MS para o PAB-Fixo^11^. Nesse modelo, os repasses *per capita* foram definidos de acordo com uma nota de 0 a 10, calculada para cada município, considerando os seguintes indicadores: produto interno bruto *per capita*, percentual da população com plano de saúde, percentual da população com Bolsa Família, percentual da população em extrema pobreza e densidade demográfica. O índice criado a partir desses indicadores reflete o grau de vulnerabilidade socioeconômica da população de cada município, em que zero indica grau máximo de vulnerabilidade, ou seja, piores condições socioeconômicas. Os municípios foram classificados em quatro grupos, em um gradiente de vulnerabilidade socioeconômica, de maior para menor:

- Grupo I: com pontuação menor que 5,3 e população de até 50 mil habitantes – mínimo de R$ 28,00 por habitante ao ano (hab/ano);
- Grupo II: com pontuação entre 5,3 e 5,8 e população de até 100 mil habitantes e os com pontuação menor que 5,3 e população entre 50 e 100 mil habitantes – mínimo de R$ 26,00 hab/ano;
- Grupo III: com pontuação entre 5,8 e 6,1 e população de até 500 mil habitantes e os com pontuação menor que 5,8 e população entre 100 e 500 mil habitantes – mínimo de R$ 24,00 hab/ano;
- Grupo IV: não contemplados nos itens anteriores e o Distrito Federal (Brasília) – mínimo de R$ 23,00 hab/ano.

Uma vez que o Ministério da Saúde não publicou a lista de municípios por grupo, a classificação de cada um deles teve que ser inferida a partir da divisão entre o valor do PAB-Fixo anual pela população de referência 2012, para o período de 2015 a 2017 (anexo II da Portaria MS/GM nº 1.409, de 2013)^12^, e entre o valor do PAB-Fixo anual pela população de referência 2016, para 2018 e 2019 (anexo II da Portaria MS/GM nº 3.947, de 2017)^13^, obtendo-se, assim, o valor do repasse *per capita* anual. Assumiu-se o pressuposto de que os valores publicados nas portarias são os resultantes da aplicação dos critérios e métodos adotados pelo órgão para a alocação equitativa de recursos do PAB-Fixo.

Duas determinações do MS quanto às transferências do PAB-Fixo foram analisadas pelo possível impacto para a inferência dos grupos. A primeira é que o ministério estabeleceu que os municípios não sofreriam redução no valor do PAB-Fixo devido à variação da população. Comparando-se o valor transferido, constante do arquivo de repasse do Fundo Nacional de Saúde (FNS) de 2018 em relação aos da Portaria MS/GM nº 3.947^13^, verificou-se que dos 455 municípios que tiveram redução da população de referência (2016 comparado a 2012), o repasse FNS, em comparação ao da portaria, foi maior para 450 municípios e igual para cinco deles. Logo, os valores da portaria parecem não conter ajustes por redução de população.

A segunda é que o MS decidiu integrar ao PAB-Fixo, em 2013, os valores da estratégia Compensação de Especificidades Regionais (CER) do PAB-Variável^11,18^. Com isso, ficou mais complicado reproduzir a comparação mencionada para os casos de redução da população de referência (2012 comparado a 2010)^13^. Contudo, quando se compara o valor *per capita* calculado a partir do valor anual do PAB-Fixo da Portaria MS/GM nº 1.409 e aquele obtido a partir dos arquivos de repasse do FNS, incluindo a estratégia CER, observa-se maior consistência no primeiro caso, com valores maiores que R$ 23,00 *per capita*/ano. O mesmo não ocorre quando se utiliza a informação do arquivo de repasse do Fundo Nacional de Saúde, pois obtêm-se valores de repasse inferiores a esse mínimo. Esse resultado indica que o valor da portaria engloba todo o valor da estratégia CER, sendo mais adequada a sua utilização na inferência dos grupos.

Os valores de incremento ao PAB transferidos pelo Ministério da Saúde a cada município, foram obtidos dos arquivos de repasse do FNS. Esse incremento diz respeito a recursos federais alocados por EP^5^. Para fins de comparação no período analisado, os recursos destinados ao PAB-Fixo e ao incremento ao Piso da Atenção Básica foram atualizados monetariamente para 2020, utilizando-se, para tanto, o Índice de Preços ao Consumidor Amplo (IPCA) anual médio.

Os dados foram organizados em planilhas eletrônicas e sumarizados com a produção de estatísticas descritivas básicas. O teste Z foi aplicado com o suporte do software RStudio, versão 2021.09.0, considerando o intervalo de confiança de 95% (IC95%), para a comparação das proporções de municípios e dos recursos entre o PAB e o seu incremento nos quatro grupos^19^.

## RESULTADOS

Em 2015, 83,6% dos municípios (n = 5.570) foram classificados nos grupos I (n = 2.604) e II (n = 2.051) quanto ao PAB-Fixo (Tabela 1 e Tabela 2). Em 2019, esse percentual cresceu (91,3%), sendo 3.958 municípios no grupo I e 1.130 no grupo II.

**Tabela 1:** Municípios favorecidos por emendas parlamentares para incremento ao Piso da Atenção Básica (PAB) entre 2015 e 2019, segundo grandes regiões e grupos de alocação de recursos do PAB-Fixo definidos pelo Ministério da Saúde.

Regiões	Grupos de alocação de recursos do PAB-Fixo								Total	

	I		II		III		IV			
				
	n	% do grupo	n	% do grupo	n	% do grupo	n	% do grupo	n	% do grupo
2015, 2016 e 2017 - Todos os municípios (PAB-Fixo)										
CO	273	10,5	162	7,9	27	3,6	5	3,0	467	8,4
NE	1.079	41,4	621	30,3	81	10,8	13	7,9	1.794	32,2
N	261	10,0	152	7,4	34	4,5	3	1,8	450	8,1
SE	617	23,7	600	29,3	348	46,3	103	62,8	1.668	29,9
S	374	14,4	516	25,2	261	34,8	40	24,4	1.191	21,4
**Total**	**2.604**	**100,0**	**2.051**	**100,0**	**751**	**100,0**	**164**	**100,0**	**5.570**	**100,0**
2015 - Municípios beneficiados com o incremento ao PAB por emendas parlamentares										
CO	0	0,0	0	0,0	0	0,0	0	0,0	0	0,0
NE	49	35,3	30	26,3	4	7,7	2	22,2	85	27,1
N	16	11,5	15	13,2	3	5,8	0	0,0	34	10,8
SE	42	30,2	30	26,3	26	50,0	5	55,6	103	32,8
S	32	23,0	39	34,2	19	36,5	2	22,2	92	29,3
**Total**	**139**	**100,0**	**114**	**100,0**	**52**	**100,0**	**9**	**100,0**	**314**	**100,0**
2016 - Municípios beneficiados com o incremento ao PAB por emendas parlamentares										
CO	14	1,6	4	0,6	4	1,2	0	0,0	22	1,1
NE	361	41,2	188	25,9	36	11,2	4	8,2	589	29,9
N	78	8,9	49	6,8	12	3,7	1	2,0	140	7,1
SE	231	26,3	215	29,7	135	42,1	30	61,2	611	31,0
S	193	22,0	269	37,1	134	41,7	14	28,6	610	30,9
**Total**	**877**	**100,0**	**725**	**100,0**	**321**	**100,0**	**49**	**100,0**	**1.972**	**100,0**
2017 - Municípios beneficiados com o incremento ao PAB por emendas parlamentares										
CO	104	6,2	60	4,4	8	1,6	2	2,2	174	4,8
NE	699	42,0	428	31,4	54	10,8	8	8,8	1.189	32,8
N	203	12,2	112	8,2	16	3,2	1	1,1	332	9,2
SE	394	23,7	391	28,7	229	45,7	54	59,3	1.068	29,5
S	265	15,9	372	27,3	194	38,7	26	28,6	857	23,7
**Total**	**1.665**	**100,0**	**1.363**	**100,0**	**501**	**100,0**	**91**	**100,0**	**3.620**	**100,0**
2018 e 2019 - Todos os municípios (PAB-Fixo)										
CO	411	10,4	33	2,9	18	4,6	5	5,3	467	8,4
NE	1.608	40,6	123	10,9	52	13,4	11	11,7	1.794	32,2
N	379	9,6	44	3,9	23	5,9	4	4,3	450	8,1
SE	957	24,2	464	41,1	190	49,0	57	60,6	1.668	29,9
S	603	15,2	466	41,2	105	27,1	17	18,1	1.191	21,4
**Total**	**3.958**	**100,0**	**1.130**	**100,0**	**388**	**100,0**	**94**	**100,0**	**5.570**	**100,0**
2018 - Municípios beneficiados com o incremento ao PAB por emendas parlamentares										
CO	298	8,6	21	2,1	9	3,0	1	1,5	329	6,8
NE	1.455	42,0	111	11,2	44	14,5	7	10,4	1.617	33,5
N	359	10,4	36	3,6	16	5,3	3	4,5	414	8,6
SE	809	23,3	381	38,6	146	48,2	40	59,7	1.376	28,5
S	544	15,7	439	44,4	88	29,0	16	23,9	1.087	22,5
**Total**	**3.465**	**100,0**	**988**	**100,0**	**303**	**100,0**	**67**	**100,0**	**4.823**	**100,0**
2019 - Municípios beneficiados com o incremento ao PAB por emendas parlamentares										
CO	367	10,0	29	2,8	14	3,9	2	2,3	412	8,0
NE	1.554	42,6	117	11,3	49	13,7	11	12,5	1.731	33,7
N	369	10,1	42	4,1	20	5,6	4	4,5	435	8,5
SE	824	22,6	410	39,7	173	48,3	56	63,6	1.463	28,5
S	538	14,7	434	42,1	102	28,5	15	17,0	1.089	21,2
**Total**	**3.652**	**100,0**	**1.032**	**100,0**	**358**	**100,0**	**88**	**100,0**	**5.130**	**100,0**

Fontes: Brasil (2013; 2017)^12,13^; e Fundo Nacional de Saúde (FNS). Arquivos de repasse. Disponíveis em: <https://portalfns.saude.gov.br/downloads/>.

**Tabela 2:** Municípios favorecidos por emendas parlamentares para incremento ao Piso da Atenção Básica (PAB) entre 2015 e 2019, segundo faixas de tamanho da população e grupos de alocação de recursos do PAB-Fixo definidos pelo Ministério da Saúde.

Faixas de tamanho da população	Grupos de alocação de recursos do PAB-Fixo								Total	

	I		II		III		IV			
				
	n	% do grupo	n	% do grupo	n	% do grupo	n	% do grupo	n	% do grupo
2015, 2016 e 2017 – Todos os municípios (PAB-Fixo)										
≤ 5.000	694	26,7	508	24,8	90	12,0	8	4,9	1.300	23,3
5.001–10.000	670	25,7	434	21,2	96	12,8	11	6,7	1.211	21,7
10.001–20.000	755	29,0	508	24,8	121	16,1	8	4,9	1.392	25,0
20.001–50.000	485	18,6	416	20,3	126	16,8	28	17,1	1.055	18,9
50.001–100.000	0	0,0	185	9,0	126	16,8	13	7,9	324	5,8
100.001–500.000	0	0,0	0	0,0	192	25,6	58	35,4	250	4,5
≥ 500.000	0	0,0	0	0,0	0	0,0	38	23,2	38	0,7
**Total**	**2.604**	**100,0**	**2.051**	**100,0**	**751**	**100,0**	**164**	**100,0**	**5.570**	**100,0**
2015 – Municípios beneficiados com o incremento ao PAB por emendas parlamentares										
≤ 5.000	36	25,9	22	19,3	4	7,7	0	0,0	62	19,7
5.001–10.000	43	30,9	27	23,7	10	19,2	0	0,0	80	25,5
10.001–20.000	33	23,7	32	28,1	10	19,2	0	0,0	75	23,9
20.001–50.000	27	19,4	26	22,8	10	19,2	3	33,3	66	21,0
50.001–100.000	0	0,0	7	6,1	7	13,5	0	0,0	14	4,5
100.001–500.000	0	0,0	0	0,0	11	21,2	2	22,2	13	4,1
≥ 500.000	0	0,0	0	0,0	0	0,0	4	44,4	4	1,3
**Total**	**139**	**100,0**	**114**	**100,0**	**52**	**100,0**	**9**	**100,0**	**314**	**100,0**
2016 – Municípios beneficiados com o incremento ao PAB por emendas parlamentares										
≤ 5.000	221	25,2	151	20,8	32	10,0	0	0,0	404	20,5
5.001–10.000	232	26,5	157	21,7	44	13,7	3	6,1	436	22,1
10.001–20.000	250	28,5	195	26,9	59	18,4	1	2,0	505	25,6
20.001–50.000	174	19,8	161	22,2	55	17,1	10	20,4	400	20,3
50.001–100.000	0	0,0	61	8,4	58	18,1	5	10,2	124	6,3
100.001–500.000	0	0,0	0	0,0	73	22,7	16	32,7	89	4,5
≥ 500.000	0	0,0	0	0,0	0	0,0	14	28,6	14	0,7
**Total**	**877**	**100,0**	**725**	**100,0**	**321**	**100,0**	**49**	**100,0**	**1.972**	**100,0**
2017 – Municípios beneficiados com o incremento ao PAB por emendas parlamentares										
≤ 5.000	485	29,1	337	24,7	62	12,4	5	5,5	889	24,6
5.001–10.000	454	27,3	289	21,2	72	14,4	7	7,7	822	22,7
10.001–20.000	457	27,4	337	24,7	94	18,8	3	3,3	891	24,6
20.001–50.000	269	16,2	274	20,1	84	16,8	18	19,8	645	17,8
50.001–100.000	0	0,0	126	9,2	84	16,8	7	7,7	217	6,0
100.001–500.000	0	0,0	0	0,0	105	21,0	29	31,9	134	3,7
≥ 500.000	0	0,0	0	0,0	0	0,0	22	24,2	22	0,6
**Total**	**1.665**	**100,0**	**1.363**	**100,0**	**501**	**100,0**	**91**	**100,0**	**3.620**	**100,0**
2018 e 2019 – Todos os municípios (recebimento do PAB-Fixo)										
≤ 5.000	1.052	26,6	166	14,7	16	4,1	3	3,2	1.237	22,2
5.001–10.000	1.018	25,7	169	15,0	22	5,7	0	0,0	1.209	21,7
10.001–20.000	1.096	27,7	246	21,8	17	4,4	5	5,3	1.364	24,5
20.001–50.000	792	20,0	248	21,9	52	13,4	9	9,6	1.101	19,8
50.001–100.000	0	0,0	301	26,6	43	11,1	6	6,4	350	6,3
100.001–500.000	0	0,0	0	0,0	238	61,3	30	31,9	268	4,8
≥ 500.000	0	0,0	0	0,0	0	0,0	41	43,6	41	0,7
**Total**	**3.958**	**100,0**	**1.130**	**100,0**	**388**	**100,0**	**94**	**100,0**	**5.570**	**100,0**
2018 – Municípios beneficiados com o incremento ao PAB por emendas parlamentares										
≤ 5.000	908	26,2	147	14,9	12	4,0	3	4,5	1.070	22,2
5.001–10.000	907	26,2	155	15,7	18	5,9	0	0,0	1.080	22,4
10.001–20.000	963	27,8	229	23,2	16	5,3	3	4,5	1.211	25,1
20.001–50.000	687	19,8	202	20,4	41	13,5	9	13,4	939	19,5
50.001–100.000	0	0,0	255	25,8	35	11,6	4	6,0	294	6,1
100.001–500.000	0	0,0	0	0,0	181	59,7	23	34,3	204	4,2
≥ 500.000	0	0,0	0	0,0	0	0,0	25	37,3	25	0,5
**Total**	**3.465**	**100,0**	**988**	**100,0**	**303**	**100,0**	**67**	**100,0**	**4.823**	**100,0**
2019 – Municípios beneficiados com o incremento ao PAB por emendas parlamentares										
≤ 5.000	931	25,5	143	13,9	13	3,6	2	2,3	1.089	21,2
5.001–10.000	935	25,6	149	14,4	17	4,7	0	0,0	1.101	21,5
10.001–20.000	1.042	28,5	225	21,8	16	4,5	4	4,5	1.287	25,1
20.001–50.000	744	20,4	230	22,3	50	14,0	9	10,2	1.033	20,1
50.001–100.000	0	0,0	285	27,6	41	11,5	6	6,8	332	6,5
100.001–500.000	0	0,0	0	0,0	221	61,7	29	33,0	250	4,9
≥ 500.000	0	0,0	0	0,0	0	0,0	38	43,2	38	0,7
**Total**	**3.652**	**100,0**	**1.032**	**100,0**	**358**	**100,0**	**88**	**100,0**	**5.130**	**100,0**

Fontes: Brasil (2013; 2017)^12,13^; e Fundo Nacional de Saúde (FNS). Arquivos de repasse. Disponíveis em: <https://portalfns.saude.gov.br/downloads/>.

No grupo 1, de maior vulnerabilidade socioeconômica, mais da metade dos municípios pertence às regiões Centro-Oeste e Nordeste, com população igual ou menor do que 50 mil habitantes (referência PAB-Fixo 2015) (Tabela 1). Em relação aos municípios beneficiados por EP, entre 2015 e 2017, a proporção dos favorecidos dessas regiões ficou abaixo de 50% para os integrantes do grupo I, sendo mais prevalentes os municípios das regiões Sudeste e Sul. Em 2018 e 2019, observa-se maior equilíbrio entre a proporção de favorecidos por grupo e regiões para o PAB-Fixo e o incremento ao PAB.

Por porte populacional, a alocação de recursos por EP se aproximou daquela definida para o Piso da Atenção Básica, no caso do grupo I (Tabela 2). Em 2015, 10,5% dos municípios com mais de 500 mil habitantes e 4,8% dos com até 5 mil habitantes foram favorecidos por EP. Em 2017 e 2019, esses percentuais ampliaram para 57,6% e 68,4%, e de 92,7% e 88,0%, respectivamente.

Entre 2015 (valor válido até 2017) e 2019 (valor válido em 2018 e 2019), verifica-se redução dos recursos alocados ao PAB-Fixo, em termos reais (Tabela 3). O PAB-Fixo passou de R$ 6,04 bilhões para R$ 5,51 bilhões a preços de 2020 (-8,8%). Enquanto o incremento ao PAB passou de R$ 95,06 milhões para R$ 5,58 bilhões, com crescimento de 5.767% no mesmo período.

**Tabela 3:** Repasses federais do PAB-Fixo aos municípios e para incremento ao PAB por emendas parlamentares, segundo os grupos de alocação de recursos do PAB-Fixo definidos pelo Ministério da Saúde.

Grupos de alocação de recursos	PAB-Fixo	Incremento ao PAB			PAB-Fixo	Incremento ao PAB	
			
	2015, 2016 e 2017	2015	2016	2017	2018 e 2019	2018	2019
Valor total em R$ de 2020							
Grupo I	1.124.748.593	34.042.736	348.535.796	888.855.288	1.501.737.158	2.806.017.353	3.159.522.314
Grupo II	1.242.968.275	32.436.990	292.125.244	760.961.497	943.886.451	833.223.227	1.104.026.427
Grupo III	1.594.723.442	19.670.932	212.915.263	381.464.102	1.317.320.727	509.097.654	888.507.783
Grupo IV	2.078.489.331	8.908.991	84.636.888	113.825.593	1.745.813.233	190.696.645	424.829.357
**Brasil**	**6.040.929.641**	**95.059.649**	**938.213.191**	**2.145.106.481**	**5.508.757.569**	**4.339.034.880**	**5.576.885.881**
Valor *per capita* médio em R$ de 2020							
Grupo I	35	31	44	61	30	81	80
Grupo II	34	27	36	50	28	45	46
Grupo III	31	13	21	30	26	18	23
Grupo IV	29	6	8	15	25	9	15
**Brasil**	**34**	**26**	**36**	**52**	**29**	**69**	**68**
Percentual de municípios beneficiados por grupo (%)							
Grupo I	100	5	34	64	100	88	92
Grupo II	100	6	35	66	100	87	91
Grupo III	100	7	43	67	100	78	92
Grupo IV	100	5	30	55	100	71	94
**Brasil**	**100**	**6**	**35**	**65**	**100**	**87**	**92**

PAB: Piso da Atenção Básica.

Fontes: Brasil (2013; 2017)^12,13^; e Fundo Nacional de Saúde (FNS). Arquivos de repasse. Disponíveis em: <https://portalfns.saude.gov.br/downloads/>.

Valores atualizados monetariamente pelo IPCA anual médio.

Em 2017, o incremento equivalia a 35,5% dos recursos do PAB-Fixo (R$ 2,15 bilhões em R$ 6,04 bilhões). Em 2019, esse percentual foi de 101,2% (R$ 5,58 bilhões em R$ 5,51 bilhões). Do valor alocado por EP (incremento) em 2015, 69,9% foram destinados aos grupos I e II (R$ 66,5 milhões em R$ 95,06 milhões). Em 2019, foram ampliados para 76,5% para os mesmos grupos (R$ 4,26 bilhões em R$ 5,58 bilhões).

Ainda na Tabela 3, verifica-se que, em 2015, 5% dos municípios do grupo I foram beneficiados, em média, com R$ 31,00 *per capita* (a preços de 2020) para incremento ao PAB. No total, receberam R$ 35,00 *per capita* do PAB-Fixo acrescidos de R$ 31,00 *per capita* por EP. No mesmo ano, 95% dos municípios desse grupo contaram apenas com o valor de R$ 35,00 *per capita* do PAB-Fixo. Em 2019, 92% dos municípios do grupo I foram favorecidos por EP e tiveram adicional ao Piso da Atenção Básica de R$ 80,00 *per capita* em média, enquanto 8% dos municípios desse grupo tiveram apenas o PAB-Fixo (R$ 30,00 *per capita*).

Na Tabela 4, comparam-se as proporções de municípios e de recursos alocados para as classes PAB-Fixo e incremento ao PAB por grupos. Verifica-se que não se pode rejeitar a hipótese nula de igualdade entre as classes quanto às proporções de municípios, uma vez que o *p-valor* é maior do que 0,05. Isso significa que, para o conjunto de beneficiados por EP, a participação dos municípios por grupo foi semelhante à participação dos municípios do PAB-Fixo por grupo.

**Tabela 4:** Proporção dos municípios e dos recursos em relação ao PAB-Fixo e ao incremento ao PAB no período de 2015 a 2019, segundo os grupos de alocação de recursos do PAB-Fixo.

Grupos de alocação de recursos	Base de comparação 2015				Base de comparação 2018			
	
	PAB-Fixo	Incremento ao PAB	Teste Z		PAB-Fixo	Incremento ao PAB	Teste Z	
					
	2015, 2016 e 2017	Média 2015–2017	X2	p	2018 e 2019	Média 2018–2019	X2	p
Proporção dos municípios								
Grupo I	47	45	0,02	0,88720	71	72	8,86	1,00000
Grupo II	37	37	0,00	1,00000	20	20	0,00	1,00000
Grupo III	13	16	0,16	0,68790	7	7	0,00	1,00000
Grupo IV	3	3	0,00	1,00000	2	2	0,00	1,00000
**Total**	**100**	**100**			**100**	**100**		
Proporção dos recursos								
Grupo I	19	38	7,95	0,00481^b^	27	61	22,10	0,002591^b^
Grupo II	21	34	3,61	0,05739	17	19	0,03	0,85400
Grupo III	26	20	0,71	0,40080	24	14	2,63	0,10480
Grupo IV	34	8	18,84	0,00001^a^	32	6	20,30	0,006601^b^
**Total**	**100**	**100**			**100**	**100**		

PAB: Piso da Atenção Básica.

Fontes: Brasil (2013; 2017)^12,13^; e Fundo Nacional de Saúde (FNS). Arquivos de repasse. Disponíveis em: <https://portalfns.saude.gov.br/downloads/>.

^a^ Valor de p < 0,001 (significância estatística ao nível de 0,1%);

^b^ Valor de p < 0,01 (significância estatística ao nível de 1%).

Entretanto, em relação às proporções de recursos, observa-se *p-valor* estatisticamente significante ao nível de 0,1%, indicando que as classes PAB-Fixo 2015 e média do incremento 2015–2017 são diferentes para o grupo IV, e ao nível de 1% para o grupo I dessas mesmas classes, e grupos I e IV das classes PAB-Fixo 2018 e média do incremento 2018–2019. Ou seja, nos recursos alocados por EP, os municípios do grupo I tiveram maior participação e os do grupo IV menor participação em relação à participação desses grupos quanto aos recursos do PAB-Fixo.

Verifica-se aumento significativo do PAB-Fixo acrescido do incremento ao PAB a partir de 2017 (Figura). Na comparação entre os grupos, para os municípios do grupo I, o valor per capita médio do PAB-Fixo com incremento passou de R$ 66,00 para R$ 108,00 (64,3%) entre 2015 e 2019. Para o grupo IV, o aumento foi de 11,8%, passando de R$ 35,00 para R$ 39,00 no mesmo período.

**Figura:** Média e mediana do Piso da Atenção Básica-Fixo (PAB-Fixo) *per capita* e do PAB-Fixo acrescido do incremento ao PAB *per capita*, segundo os grupos de alocação de recursos do PAB-Fixo aos municípios, definidos pelo Ministério da Saúde. Brasil, 2015–2019.

Fontes: Brasil (2013; 2017)^12,13^; e Fundo Nacional de Saúde (FNS). Arquivos de repasse. Disponíveis em: <https://portalfns.saude.gov.br/downloads/>.

Elaboração própria.

Ainda na Figura, verifica-se que as médias e as medianas do PAB-Fixo apresentam baixa variação, o que não ocorre quando são somados os valores do incremento. Isso indica que há municípios com incremento ao PAB *per capita* bastante alto, o que faz com que haja maior distanciamento entre a média e mediana dos valores analisados. Por fim, destaca-se a redução, em termos reais, das médias e das medianas *per capita* do PAB-Fixo. Na média, considerando todos os municípios, passou de R$ 34,00, em 2015, para R$ 29,00, em 2019 (-14,7%).

## DISCUSSÃO

Algumas metodologias vêm sendo desenvolvidas no âmbito dos sistemas de saúde para a alocação equitativa de recursos^20^. Nesse sentido, diferentes acepções de equidade têm sido utilizadas, tais como: i) a completa equalização de oportunidades de acesso à mesma quantidade de serviços, com relação às necessidades; ii) a garantia de que nenhum grupo em particular fique em desvantagem; e iii) de que todos tenham oportunidade igual a uma vida saudável^21^.

A equidade também é citada como oportunidade justa para todos, igualdade de acesso a serviços de saúde com base nas necessidades, e ausência de desigualdades sistemáticas em saúde entre grupos socioeconomicamente diferentes, sendo reportada na literatura como o critério mais utilizado pelos tomadores de decisão sobre a alocação de recursos em saúde^22^.

No Brasil, a ideia de alocação equitativa de recursos está intrinsecamente associada à de necessidades de saúde. No geral, os autores defendem a centralidade e reconhecem a forma complexa do conceito de necessidades de saúde para a alocação de recursos, contudo, não o explicitam. Mas é possível presumir o seu alcance para além das fronteiras do sistema, em razão do uso de indicadores socioeconômicos, demográficos e de saúde nas metodologias propostas ou analisadas^23^.

As metodologias para a alocação equitativa de recursos traduzem, geralmente, a ideia de que é preciso considerar as desiguais condições de vida da população, a fim de destinar recursos de forma desigual. O propósito é destinar mais recursos para os grupos mais desfavorecidos dos pontos de vista demográfico, social, econômico e de saúde.

No SUS, a implantação do PAB-Fixo está entre as iniciativas adotadas pelo MS para promover a redução das desigualdades regionais por meio de uma alocação desigual dos recursos federais para a saúde^26^. Ainda que a iniciativa possa ser eventualmente criticada quanto ao método adotado e aos resultados obtidos, não se pode desconhecer seu mérito ao introduzir a ideia da alocação equitativa de recursos no SUS. Esse sistema apresenta um padrão de responsabilidade compartilhada entre os entes no financiamento de serviços de atenção básica. Entretanto, cabe ao Ministério da Saúde a distribuição de recursos financeiros visando compensar desigualdades entre os municípios; principais responsáveis pela gestão desses serviços^9^.

Este estudo demonstra que, em termos de municípios favorecidos, as EP acompanharam os grupos de alocação definidos pelo MS, beneficiando-os em proporção semelhante à distribuição feita para o PAB-Fixo. Contudo, o PAB-Fixo favorece a todos os municípios, diferentemente das EP, ainda que a sua cobertura tenha aumentado no período analisado.

Quanto à proporção de recursos alocados, observa-se, em relação ao modelo de alocação do PAB-Fixo, que os recursos das EP foram destinados em maior proporção para os municípios do grupo I e em menor para os do grupo IV. Ou seja, foram priorizados com os aportes por EP os municípios com população de até 50 mil habitantes, mais vulneráveis socioeconomicamente, em detrimento dos municípios menos vulneráveis, com população acima de 50 mil habitantes.

Com isso, há um maior distanciamento entre os valores *per capita* do PAB quando acrescidos os recursos de incremento. Populações de municípios menores estão sendo beneficiadas com muito mais recursos para o financiamento da APS, que as de municípios maiores, menos vulneráveis.

Em princípio, tal situação é desejável quando se fala em alocação equitativa. Contudo, é preciso ponderar sobre a conjuntura atual do financiamento do SUS e os possíveis impactos da alocação de recursos por EP, dada a grande restrição orçamentária imposta ao orçamento do ministério pelo teto de gastos para as despesas primárias da União e congelamento da aplicação mínima federal em ações e serviços públicos de saúde^27^. Como as despesas por EP são contabilizadas na aplicação mínima, maior alocação de recursos por EP reduz a parcela de alocação própria pelo Ministério da Saúde em ações e serviços e pode implicar a realocação de recursos de outras áreas. No caso analisado, de EP para incremento ao PAB.

Também é importante destacar que mesmo entre os municípios do grupo I, os beneficiados por EP receberam muito mais recursos *per capita* que os não beneficiados. Com isso, as emendas acabaram gerando tratamento desigual entre os mais vulneráveis.

O valor *per capita* muito diferente entre os grupos de municípios, implica benefício diferenciado entre as suas populações. As ações e serviços públicos de saúde devem ser garantidos em todos os municípios e, naqueles menores, os custos de sua oferta geralmente são maiores^28^, mas é preciso lembrar que há dificuldades para estruturá-los nas periferias de cidades de médio e grande porte populacional^29^. Com a crise financeira de 2014 a 2016, e mais recentemente com os impactos da pandemia sobre a economia brasileira, houve queda da arrecadação dos municípios e, com isso, maiores dificuldades para a alocação de recursos à saúde. Os municípios já os aplicam muito acima do percentual mínimo obrigatório^30^.

Ao final, uma alocação de recursos federais que não leve em consideração a capacidade fiscal diferenciada dos entes, também pode ser causa de iniquidade, ainda que seu objetivo seja a equidade. Para mitigar esse tipo de problema, a adoção de critérios técnicos tem sido apontada como uma necessidade^31^. Essa é uma questão que precisa ser aprofundada em estudos futuros para o caso das EP de forma geral.

Outras questões que devem ser consideradas é que as EP estão sendo utilizadas na garantia da base de sustentação do governo federal, no Congresso Nacional, de uma forma inédita, considerando o elevado montante de recursos envolvidos, e que há grande deficit de transparência na sua execução. Essa falta de transparência, especialmente das EP de relator, foi questionada no Supremo Tribunal Federal (STF), que determinou ao Congresso a publicação da lista de favorecidos e parlamentares, além dos valores alocados^32^.

As consequências de uma alocação de recursos que considere critérios estritamente políticos podem ser muito negativas para o SUS, dada a conjuntura recente de restrição orçamentária já mencionada. Além de provocar maiores desigualdades, pode ser mais ineficiente, o que é inaceitável diante da limitação de recursos para financiamento da saúde pública no país.

Sobre este trabalho, aponta-se como limitação a inferência dos grupos de alocação de recursos, o que gera alguma incerteza sobre a categoria de cada município. A falta de transparência sobre o método empregado pelo Ministério da Saúde constitui barreira não apenas para o conhecimento da classificação desses entes, como para qualquer estudo que almeje investigar esta iniciativa de alocação equitativa de recursos.

Para finalizar, destaca-se que se analisaram tão somente as implicações das EP sobre o modelo adotado pelo ministério para o PAB-Fixo. O modelo em si não foi avaliado. A conclusão é de que as EP distorceram o modelo de alocação equitativa de recursos pensado pelo MS para o PAB-Fixo, ao destinar recursos em proporção muito maior para os municípios do grupo I e muito menor para os do grupo IV, em desacordo com esse modelo, e por não beneficiar a todos os municípios, nem mesmo os mais vulneráveis.
